# The Impact of Soybean Genotypes on Rhizosphere Microbial Dynamics and Nodulation Efficiency

**DOI:** 10.3390/ijms26072878

**Published:** 2025-03-21

**Authors:** Doni Thingujam, Aqsa Majeed, Bala Subramanyam Sivarathri, Nisarga Kodadinne Narayana, Mohan K. Bista, Katie E. Cowart, Adelle J. Knight, Karolina M. Pajerowska-Mukhtar, Raju Bheemanahalli, M. Shahid Mukhtar

**Affiliations:** 1 Department of Biological Sciences, Clemson University, 132 Long Hall, Clemson, SC 29634, USA; dthingu@clemson.edu (D.T.); kmukhta@clemson.edu (K.M.P.-M.); 2 Department of Biology, University of Alabama at Birmingham, 3100 East Science Hall, 902 14th Street South, Birmingham, AL 35294, USA; amajeed@clemson.edu (A.M.); kecowart@uab.edu (K.E.C.); addiekni@uab.edu (A.J.K.); 3 Department of Genetics & Biochemistry, Biosystems Research Complex, Clemson University, 105 Collings St., Clemson, SC 29634, USA; 4 Department of Plant and Soil Sciences, Mississippi State University, Mississippi State, MS 39762, USA; bs2441@msstate.edu (B.S.S.); nk581@msstate.edu (N.K.N.); mkb621@msstate.edu (M.K.B.)

**Keywords:** stomata, microbiome, amplicon sequencing, taxonomic abundance, alpha diversity, beta diversity

## Abstract

Rhizosphere microbiome exerts a significant role in plant health, influencing nutrient availability, disease resistance, and overall plant growth. Establishing a robust and efficient nodulation process is essential for optimal nitrogen fixation in legumes like soybeans. Different soybean genotypes exhibit variations in their rhizosphere microbiome, potentially impacting nitrogen fixation through nodulation. However, a detailed understanding of how specific soybean genotypes influence rhizosphere microbial communities and nodulation patterns remains limited. Our study aims to investigate the relationship between rhizosphere microbial abundance and plant growth in four soybean genotypes. We evaluated plant growth parameters, including biomass, leaf area, and stomatal conductance, and identified significant genotypic differences in nodulation. Specifically, genotypes PI 458505 and PI 603490 exhibited high levels of nodulation, while PI 605839A and PI 548400 displayed low nodulation. 16S rRNA gene amplicon sequencing revealed diverse bacterial communities in the rhizosphere, with *Proteobacteria* as the dominant phylum. High-nodulation genotypes harbored more diverse microbial communities enriched with *Actinobacteria* and *Acidobacteriota*, while low-nodulation genotypes showed higher abundances of *Firmicutes* and *Planctomycetota*. Alpha and beta diversity analyses confirmed distinct microbial community structures between high- and low-nodulation groups. Our findings suggest that the rhizosphere microbiome significantly influences soybean growth and nodulation, highlighting the potential for genotype-driven strategies to enhance plant-microbe interactions and improve soybean productivity.

## 1. Introduction

The rhizosphere, the soil region surrounding plant roots, is a microenvironment where plants actively interact with a diverse community of soil microorganisms [1]. These interactions play a vital role in influencing plant growth, nutrient uptake, and ecosystem functionality [2,3]. Moreover, it has been reported that the rhizosphere microbiome significantly contributes to plant health and resilience, with microbial communities influenced by environmental factors, root exudates, and plant genotypes [4]. Among crop species, soybean (*Glycine max*) stands out as a vital focus of research due to its dual role as a global source of protein and oil, as well as its critical contribution to sustainable agricultural practices [5,6]. Soybean can establish symbiotic relationships with different nitrogen-fixing bacteria, including *Bradyrhizobium* species [7], leading to the formation of nodules—specialized structures where biological nitrogen fixation occurs [8]. This process not only reduces the reliance on synthetic nitrogen fertilizers but also enhances soil fertility, thereby positioning soybean cultivation as a key solution to global food security and environmental challenges [9]. However, the efficiency of nodulation and nitrogen fixation is not uniform across all soybean genotypes [10]. Variations in genotype can affect the recruitment of specific microbial taxa, which in turn influences vital processes such as nitrogen fixation and nodulation [1]. These genotype-driven differences might hold the potential to impact soybean productivity, particularly under diverse environmental conditions. However, the influence of rhizosphere microbial communities in the nodulation process of various soybean genotypes remains insufficiently understood.

To explore the microbial diversity associated with rhizospheres, 16S rRNA gene amplicon sequencing, a high-throughput molecular technique, has been utilized extensively [11]. This method can amplify hypervariable regions of the highly conserved 16S rRNA gene, allowing for the identification and classification of bacterial taxa, including those that are difficult to culture [12,13]. This approach can comprehensively profile bacterial communities to identify taxa associated with soil and plants and uncover potential functional roles of the rhizosphere microbiome in nutrient cycling, plant growth, and stress resilience. This sequencing technique provides a powerful tool to elucidate the complex interactions between plant and rhizosphere microbial communities [14]. Previous studies have highlighted the role of rhizosphere microbial communities in nodulation and plant growth [15]. It has also been demonstrated that different soybean genotypes host distinct microbial assemblages that contribute to variations in nodulation efficiency [16]. Similarly, genotype-specific recruitment of beneficial microbes has been highlighted for optimizing legume yield [17]. Despite these insights, there remains a need to elucidate the precise networks between genotype, microbial associations, and symbiotic effectiveness, which could guide the development of genotype-informed agricultural strategies.

Our primary objective is to address these gaps by investigating the relationship between soybean genotype, rhizosphere microbial diversity, and nodulation efficiency. Specifically, we focus on four soybean genotypes—PI 458505, PI 603490, PI 605839 A, and PI 548400—that represent diverse genetic backgrounds. First, we conducted an extensive phenotypic analysis to investigate the physiological characteristics of these four genotypes. Through amplicon sequencing, we aim to comprehensively profile rhizosphere microbial communities and correlate their composition and diversity with nodulation and plant growth parameters. Our study also emphasizes identifying microbial taxa associated with enhanced nodulation and exploring the feedback mechanisms between soybean genotypes and their rhizosphere microbiomes. Our findings hold significant potential for advancing sustainable agricultural practices by uncovering genotype-specific traits and microbial resources to enhance soybean productivity and soil health.

## 2. Results

### 2.1. Physiological Responses of Different Soybean Genotypes

**Figure 1:**
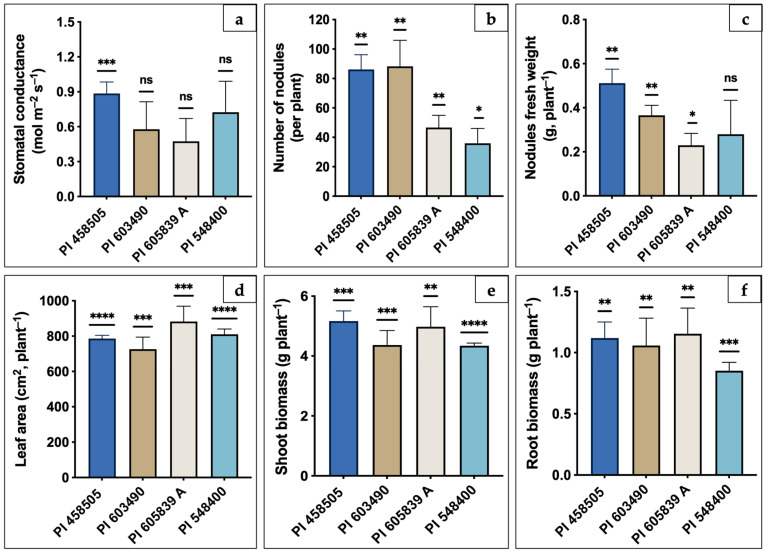
Plant physiological responses of four soybean genotypes (PI 458505, PI 603490, PI 605839 A, and PI 548400) under control growth conditions. (**a**) Stomatal conductance. (**b**) Number of nodules. (**c**) Nodule fresh weight. (**d**) Leaf area. (**e**) Shoot biomass. (**f**) Root biomass. The bars represent the mean ± standard error (SE) of five biological replicates. Statistical significance was determined using a one-sample t-test and the Wilcoxon test. Asterisks above the bars indicate statistical significance with the following *p*-value thresholds: * *p* < 0.05, ** *p* < 0.005, *** *p* < 0.0005, and **** *p* < 0.00005. ‘ns’ indicates non-significant differences (*p* ≥ 0.05).

Gaseous exchange in leaves is a crucial physiological process that optimizes CO_2_ assimilation while regulating water loss through transpiration. Stomatal conductance, a key indicator of this process, is closely tied to photosynthetic rate. In our study, significant differences in stomatal conductance were observed among the four selected soybean genotypes. PI 458505 demonstrated the highest stomatal conductance (0.89 mol m^−2^ s^−1^), indicative of superior gas exchange potential, while PI 605839A exhibited the lowest rate (0.47 mol m^−2^ s^−1^), suggesting a more conservative water-use strategy (Figure 1a). In terms of nodulation, a critical factor for biological nitrogen fixation, substantial variability was evident among the genotypes. The number of nodules per plant ranged widely, from a minimum of 36 in PI 548400 to a maximum of 86 in PI 458505, with an average of 64 nodules per plant across the genotypes. Notably, PI 458505 and PI 603490 were identified as high-nodulation genotypes, producing significantly more nodules than PI 605839A and PI 548400, classified as low-nodulation genotypes (Figure 1b). Based on the difference in the nodulation potential, the genotypes were categorized into two groups for further microbial abundance exploration (Table 1). This classification provides a framework for investigating the relationship between nodulation potential and rhizosphere microbial abundance using amplicon sequencing. This approach was considered to gain valuable insights into genotype-driven differences in microbial communities and their potential influence on plant-microbe interactions. Similarly, the fresh weight of nodules varied significantly, with PI 458505 recording the highest nodule fresh weight (0.51 g) and PI 548400 the lowest (0.28 g) (Figure 1c). Moreover, we have also measured the leaf area, an essential parameter for capturing light and facilitating photosynthesis, and differed significantly across genotypes (Figure 1d). PI 605839A exhibited the largest leaf area (883 cm^2^), indicative of greater photosynthetic potential, while PI 603490 had the smallest leaf area (726.5 cm^2^). These variations reflect inherent differences in the growth strategies of the genotypes. Biomass production also showed genotype-specific variations (Figure 1e,f). These differences further highlight the unique growth patterns of the studied genotypes. Notably, the root-to-shoot ratio under control conditions was lowest in PI 548400 (0.20), suggesting a relatively greater allocation of resources to aboveground growth compared to root development.

### 2.2. Evaluating Microbial Diversity in Soybean Rhizosphere Through Taxonomic Annotation

**Figure 2:**
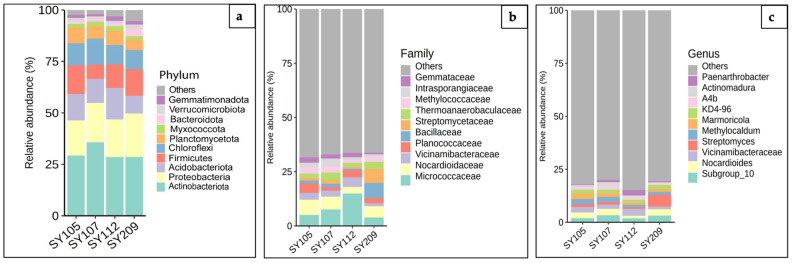
Relative abundance of bacterial communities in soybean rhizosphere samples. Bar graphs illustrate the microbial composition across four soybean rhizosphere samples (SY105, SY107, SY112, and SY209) at different taxonomic levels: (**a**) phylum, (**b**) family, and (**c**) genus. The y-axis represents the relative abundance (%) of each bacterial group. Each bar represents a sample, with different colors indicating different bacterial taxa. The phylum-level graph (**a**) shows the overall distribution of major bacterial groups. The family-level graph (**b**) provides a more detailed view of the microbial community composition, and the genus-level graph (**c**) presents the relative abundance of bacterial genera, illustrating the variations in microbial community structure and richness among the different soybean genotypes.

To analyze the microbial diversity and composite abundance among the rhizosphere samples, the DNA libraries prepared from the soil samples, including SY105, SY107, SY112, and SY209 of all four soybean genotypes, were subjected to 16S amplicon sequencing. With the taxonomic analysis of the 16S region of the amplicon sequence samples, the distribution of diverse microbial phyla, including 10 known and other Amplicon Sequence Variants (ASVs), was observed (Figure 2a). Among these ASVs, taxonomic analysis of the rhizosphere microbial communities associated with four soybean genotypes revealed a diverse and dynamic ecosystem. *Proteobacteria* consistently emerged as the dominant group across all samples at the phylum level, indicating their crucial role in rhizosphere processes. However, the relative abundances of other phyla, such as *Actinobacteria*, *Acidobacteriota*, and *Firmicutes*, varied significantly among the genotypes, suggesting distinct microbial community structures associated with each soybean genotype. Moreover, a small percentage of *Myxococcota*, *Bacteroidota*, *Verrucomicrobiota*, *Gemmatimonadota*, and other phyla were also observed. Further, the taxonomic annotations were performed at the family level, and among all the samples, about 30 to 35% of 10 known bacterial families, including *Micrococcaceae*, *Nocardioidaceae*, *Vicinamibacteraceae*, *Planococcaceae*, *Bacillaceae*, *Streptomycetaceae*, *Thermoanaerobaculaceae*, *Methylococcaceae*, *Intrasporanglaceae*, *Gemmataceae* were identified, largely followed by around 75% ASVs for “others” families (Figure 2b). For in-depth characterization of genera abundance, the annotations were further carried out at the genus level (Figure 2c). A diverse distribution of microbial genera, including *Paenarthrobacter*, *Actinomadura*, *A4b*, *KD4-96*, *Marmoricola*, *Methylocaldum*, *Streptomyces*, *Vicinamibacteracea*, *Nocardioides*, and *Subgroup_10 has been shown* across all the soybean rhizosphere samples. Additionally, we also observed a substantial proportion of the microbial community clustered within the ‘Others’ category, indicating the presence of a diverse array of genera at relatively low abundances.

### 2.3. Differential Abundance Analysis Comparing High and Low-Nodulation Soybean Groups

**Figure 3:**
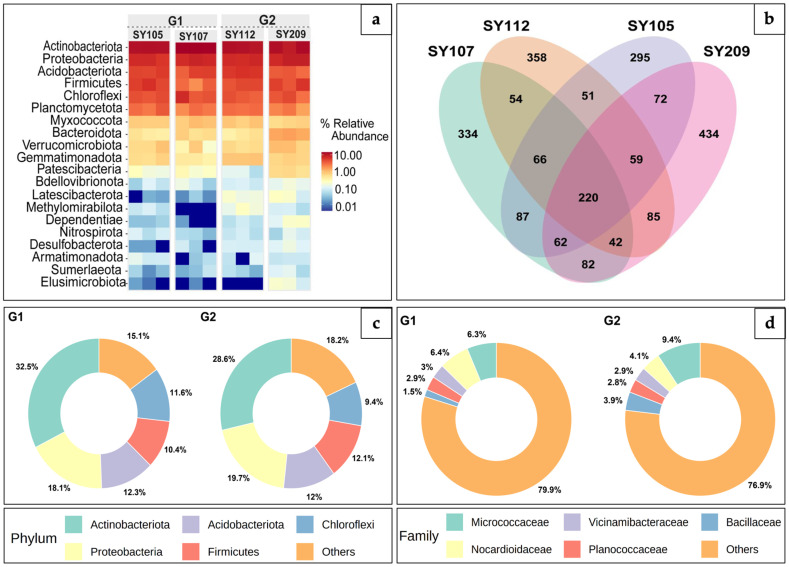
Comparative analysis of bacterial communities across soybean genotypes. (**a**) Bacterial Phyla Relative Abundance: Heatmap depicts the relative abundance of bacterial phyla in the rhizosphere of four soybean genotypes (SY105, SY107, SY112, SY209), categorized into two groups, G1 (SY105, SY107) and G2 (SY112, SY209). The color gradient indicates the differential abundance of each phylum, with darker red representing higher relative abundance and darker blue representing lower relative abundance. The phyla are listed on the left, and the samples are shown across the top. (**b**) Shared and unique OTUs: Venn diagram illustrates the overlap and unique operational taxonomic units (OTUs) among the rhizosphere microbiomes of the four soybean genotypes. The numbers within each section represent the quantity of shared or unique OTUs, providing a visual representation of community similarity and uniqueness. (**c**) Phylum relative abundance and (**d**) family relative abundance: Donut plots illustrating the relative abundance of major bacterial phyla and families, respectively, in the rhizosphere of G1 and G2. Each segment of the donut plot represents a phylum (**c**) and family (**d**), with the size of the segment corresponding to its relative abundance percentage. The legend below the plot indicates the phyla and families associated with each color.

To investigate the potential significance of bacterial phyla and family predominant in the analyzed samples, we subsequently performed the comparative study of relative abundance within and between the genotype groups. The heatmap demonstrated, for all genotypes and groups, that *Proteobacteria* were consistently the most prevalent phylum (Figure 3a). However, significant variations in the relative abundance of other phyla were observed. *Firmicutes* and *Planctomycetota* were enriched in the rhizosphere of plants from the G2, whereas *Actinobacteria* and *Acidobacteriota* were typically more prevalent in the rhizosphere of plants in the G1. In both the high-nodulation and low-nodulation groups, elucidation of the bacterial population in the rhizosphere of soybean plants showed clear trends in the relative abundance of different bacterial phyla across different rhizosphere samples. These results indicated that there might be a relationship between soybean nodulation effectiveness and the relative abundance of particular bacterial phyla. Understanding the overlapping communities and the dynamics of interactions between the different samples is essential. A complex interaction between common and distinct microbial populations was revealed by the Venn diagram, which was constructed to illustrate the rhizosphere microbial communities associated with four soybean genotypes (Figure 3b). Although the rhizosphere microbiomes of the four plants varied, they all shared a core community of 220 microbial species. In terms of the number of distinct microbial species, SY209 exhibited the most (434), whereas SY105 had the lowest number (295). Microbial communities shared by different genotypes are indicated by the overlapping sections in the Venn diagram. According to these results, the rhizosphere microbiome’s composition might be influenced by plant-specific characteristics, but there is also a core community that might be essential to plant health and ecosystem function, including soybean nodulation. The formation and activity of rhizobia, the bacteria that produce nitrogen-fixing nodules on soybean roots, might be influenced by the presence of particular microbial taxa within these distinct and shared communities. Notably, investigation of the rhizosphere microbial communities in high- and low-nodulation groups revealed distinct differences, suggesting potential correlations with soybean nodulation efficiency (Figure 3c,d). While Proteobacteria were prevalent in both groups, G1 demonstrated greater abundances of phyla *Actinobacteriota* and *Acidobacteriota*, whereas G2 exhibited higher abundances of *Firmicutes* and *Proteobacteria*. Additionally, family-level analysis revealed the association of *Micrococcaceae* and *Bacillaceae* with G2, while G1 was enriched in *Nocardioidaceae* and *Vicinamibacteraceae*. These variations in microbial associations with specific genotypes could affect the nodulation process in soybeans. Furthermore, both groups displayed a high percentage of ‘Others’ family, highlighting the presence of a diverse community of low-abundance microbial species. This result provides valuable insights into microbial distribution, emphasizing the variations between soybean genotypes.

### 2.4. Alpha (α) and Beta (β) Diversity Analysis

**Figure 4:**
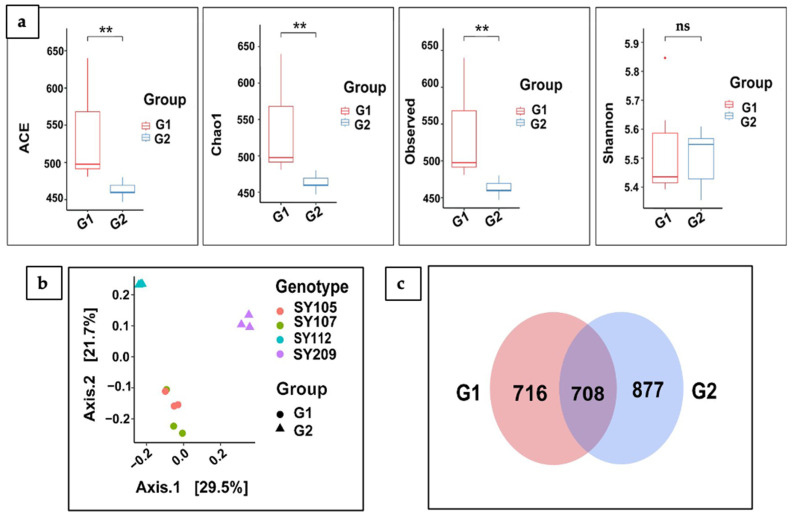
Differences in rhizosphere microbial community diversity. (**a**) Alpha diversity: Box plots illustrating the alpha diversity indices (ACE, Chao1, Observed OTUs, and Shannon) of the rhizosphere microbiomes associated with the two soybean groups, G1 (SY105, SY107) and G2 (SY112, SY209). Each box represents the interquartile range (IQR) of the data, with the median value indicated by a horizontal line. Outliers are represented by individual dots. Different colors represent each group. Asterisks (**) indicate statistically significant differences between groups, and ‘ns’ indicates non-significant differences (*p* ≥ 0.05). (**b**) Beta diversity: Principal coordinates analysis (PCoA) plot illustrating the beta diversity, or community dissimilarity, between the rhizosphere microbiomes of G1 and G2. Each point represents a single rhizosphere sample, colored by its corresponding group (G1 or G2) and shaped by its soybean genotype (SY105, SY107, SY112, SY209). Proximity of points indicates greater community similarity. (**c**) Shared and unique OTUs: Venn diagram illustrates the overlap and unique OTUs between the rhizosphere microbiomes of G1 and G2. Numbers within each section represent the number of shared or unique OTUs, providing a visual representation of community similarity and uniqueness.

We examined the alpha and beta diversities of bacterial communities in the rhizosphere, and significant changes between G1 and G2 were noted. Using a Kruskal–Wallis Rank Sum Test, alpha diversity analysis showed that the high-nodulation group and the low-nodulation group had significantly different microbial community richness and diversity (Figure 4a). G1 indicated considerably greater levels of all three alpha diversity indices (ACE, Chao1, and Observed OTUs) than G2 (*p*-values < 0.05). This suggests a more varied and abundant microbial community occurs in the rhizosphere of plants in the high-nodulation group than in those in the low-nodulation group. Although the difference was not statistically significant, the Shannon index, which considers both richness and evenness, also tended to have higher values in G1. These results imply that the composition of the rhizosphere microbial community might represent a significant factor affecting soybean nodulation efficiency.

We performed a PERMANOVA study that employs a principal coordinate study (PCoA) for beta diversity and showed a notable difference between the microbial communities linked to G1 and G2 (Figure 4b). The microbial communities in G1 and G2 are considerably different, as indicated by the plot’s clear clustering of samples based on the group membership (*p*-value = 0.005, PERMANOVA, pseudo-F test statistic). Furthermore, within each group, the PCoA map shows variations between the microbial communities related to various genotypes of soybeans. This suggests that the rhizosphere microbiome might be shaped by both plant genotype and group membership. It is evident from these observations that it is important to consider both genotype and group effects when examining the organization of the microbial community in relation to soybean nodulation. In addition, a significant overlap in the microbial communities associated with the high-nodulation group and low-nodulation group was found through the Venn diagram (Figure 4c). Both groups shared 708 identified microbial taxa, suggesting that both high- and low-nodulation conditions share a core microbiome. Furthermore, group G2 had 877 unique taxa, compared to 716 unique taxa in Group G1. These results demonstrate that each nodulation group has unique microbial signatures, even if there is a significant overlap in the microbial profile.

## 3. Discussion

The relationship between plant genotype and rhizosphere microbial communities is crucial for improving crop productivity and resilience. As a key crop in global agriculture, soybeans have distinct genotype-specific traits that influence their interaction with the rhizosphere microbiome, which in turn impacts nodulation and plant growth [18,19]. The rhizosphere, being a hotspot for microbial activity, plays an essential role in nutrient cycling, pathogen suppression, and the establishment of beneficial symbioses [1,20,21]. To elucidate the impact of soybean genotypes on nodulation, our study assessed genotype-specific traits and characterized their associated rhizosphere microbiomes, providing insights into the intricate relationship between these factors and nodulation efficiency. In our study, a distinct variation in plant traits, such as stomatal conductance, nodulation, leaf area, and biomass, was observed among the four soybean genotypes. These differences highlight their discrete growth strategies and the influence of these traits on plant-microbe interactions. Notably, PI 458505 exhibited superior stomatal conductance and nodulation capacity, suggesting enhanced gas exchange efficiency and biological nitrogen fixation potential. These findings are consistent with previous reports that emphasize the relevance of physiological traits in determining plant-microbe relationships [22,23,24].

In this study, we explored the microbial abundance in the soybean rhizosphere using 16S amplicon sequencing. Alpha diversity analysis revealed significantly higher microbial richness and diversity in the high-nodulation group compared to the low-nodulation group. This aligns with studies suggesting that diverse microbial communities provide broader functional capabilities, such as nutrient cycling, pathogen suppression, and plant growth promotion, which indirectly benefit nodulation [25]. Beta diversity analysis using PCoA demonstrated a clear separation between the microbial communities associated with G1 and G2, indicating that genotype-specific factors influence rhizosphere microbiome structure, as observed in prior studies [26,27]. Higher alpha diversity indices in G1 suggest that a more diverse microbial community creates a conducive environment for effective rhizobia colonization and symbiosis. The distinct clustering observed in beta diversity analysis supports the idea that genotype drives microbial community composition, aligning with concepts of host-driven selection for nutrient acquisition and stress tolerance [28]. The taxonomic analysis revealed the predominance of phylum *Proteobacteria* across all samples, showing their pivotal role in the rhizosphere ecosystem. *Proteobacteria* are known to engage in complex plant-microbe interactions, including support in root health, production of phytohormones, and biocontrol agents that enhance plant resilience to environmental stress interactions [1]. However, we also observed significant differences in the relative abundance of other phyla, such as *Actinobacteriota* and *Firmicutes*. *Actinobacteria* are a broad group of Gram-positive bacteria that are known to use a variety of ways to promote plant growth. These bacteria promote plant growth by producing phytohormones, such as auxins, cytokinins, and gibberellins, which regulate root development, seed germination, and overall plant health [29]. Additionally, they enhance soil health by producing siderophores, which aid in nutrient acquisition, and by decomposing organic matter [30]. Furthermore, by generating antimicrobial chemicals that shield plants from soil-borne illnesses, *Actinobacteria* aid in the suppression of pathogens [31]. *Actinobacteria* can also establish advantageous connections with plant roots, improve nitrogen cycling, and increase soil fertility [32]. One of the approaches through which *Actinobacteria* induces plant growth-promoting effect is through influencing nodulation [33]. Moreover, *Actinobacteria* have also been used to enhance the nodulation of soybeans when cocultured with *Bradyrhizobium japonicum* [34].

Conversely, we detected the enrichment of *Firmicutes* in the low-nodulation genotypes, which might suggest the potential existence of non-rhizobia in the rhizosphere, consistent with findings by Lu and colleagues [35]. Interestingly, another study by Okuba and co-workers has found an abundance of *Firmicutes* in non-nodulated (Nod(−)) and hypernodulated (Nod(++)) than wild-type nodulated (Nod(+)) soybeans [36]. Our taxonomic analysis at the genus level revealed distinct microbial signatures associated with each group. We found genera such as *Paenarthrobacter* and *Actinomadura* were enriched in high-nodulation genotypes. *Paenarthrobacter* has been shown to possess higher plant growth-promoting and phytopathogen control abilities [37]. *Paenarthrobacter nitroguajacolicus* PP3, particularly, has been found to possess higher phosphorous, potassium solubilization, and nitrogen-fixing activity [38]. In a fascinating study on soybean, a comparative evaluation of biochar and *Paenarthrobacter* sp. AT5 for reducing atrazine risks to soybeans in black soil demonstrated the greater efficiency of *Paenarthrobacter* sp. AT5 in reducing atrazine risks to soybeans than biochar. It effectively degrades atrazine in soil in comparison to biochar, thereby enhancing atrazine degradation in key growth parameters in soybeans exposed to atrazine [39]. In line with our observations, other studies have also shown the prevalence of *Actinomadura* in different plant tissues such as barley, wheat, rice, soybean, cowpea, chickpea, banana, tomato, and medicinal plants either as an epiphyte or endophyte [31,40,41]. *Actinomadura* has been shown to induce increased plant growth, total dry weight, and mineral composition of soybean when inoculated in soil [42]. This plant growth-promoting effect might be the result of their inherent property to produce organic acids such as lactic and citric acids along with distinctive activities including IAA and siderophore production, P and K solubilization, and N-fixation [43,44,45]. The consistent presence of *Micrococcaceae* across all samples highlights their critical roles in nutrient cycling and rhizobia colonization [46,47]. Nevertheless, in our study, it was observed that both shared and unique microbial taxa were present among the low- and high-nodulation groups, suggesting the existence of a core microbiome that supports essential rhizosphere functions, while unique taxa may drive genotype-specific interactions and nodulation outcomes. In our analysis, *Vicinamibacteraceae* and *Nocardioidaceae* are core microbiome families consistently shared across all four soybean genotypes, signifying their essential contributions to soil ecosystem functions. *Vicinamibacteraceae* is known for its role in polysaccharide degradation and improving soil structure [48,49]. They are also suggested to possess potential disease-suppressing agents, as their presence has been negatively correlated with the occurrence of diseases [50]. *Nocardioidaceae* has been found to produce siderophores, which may aid in plant nutrient uptake, and indole-3-acetic acid, potentially promoting plant growth. Furthermore, *Nocardioides* endophytes from wheat roots have demonstrated antifungal activity against various plant pathogens, reducing disease symptoms and benefiting plant health. These findings indicate that certain *Nocardioidaceae* members are part of the rhizosphere microbial community and can colonize root tissues as neutral or plant growth-promoting endophytes [51,52]. Interestingly, *Nocardioidaceae*, along with other bacterial families such as *Sphingomonadaceae*, *Gemmatimonadaceae*, *Xanthobacteraceae*, *Chitinophagaceae*, *Oxalobacteraceae*, and *Streptomycetaceae*, were observed to be enriched in the rhizospheres of the high-oil cultivars of soybean [53]. The persistence of these families across various soybean genotypes suggests a stable and resilient microbiome that supports nutrient cycling and disease suppression, regardless of nodulation status. Further metagenomic and metatranscriptomic analyses could provide deeper insights into the functional roles of these taxa, paving the way for microbial-based strategies to improve soybean productivity and sustainability under varying environmental conditions.

A limitation of this study is the oversight of factors that can greatly affect the composition and function of the rhizosphere microbiome, such as soil type, temperature, moisture levels, and the influence of other biotic and abiotic stresses, including drought. Previous studies have shown that drought stress negatively affects plant growth and productivity. However, plant-associated microbiomes can enhance drought resilience. Root-associated microbes, including plant growth-promoting bacteria (PGPB) and fungi (PGPF), promote plant stress tolerance through various mechanisms, such as modulating stress-related pathways, producing osmolytes, and altering gene expression to enhance water retention and nutrient uptake. There is often an enrichment of beneficial Actinobacteria in the rhizosphere under water-deficient stress. These bacteria contribute to plant health by synthesizing stress-related compounds, including glycerol-3-phosphate. Furthermore, microbial consortia, such as SPMX (a four-species bacterial community), can synergistically enhance drought tolerance by stabilizing root microbiome diversity and promoting biofilm formation, which helps retain moisture. These interactions highlight the potential of microbiome engineering for sustainable agriculture under water-scarce conditions [54,55,56]. Recent studies on soybean microbiomes have also revealed significant insights into drought stress resilience. A study conducted under extreme heat and drought conditions found that bacterial genera like Pseudomonas and *Pantoea* predominated in surviving soybean roots, while Streptomyces was more common in non-surviving roots. Beneficial bacterial strains from genera such as Acinetobacter, Pseudomonas, Enterobacter, and Stenotrophomonas have also been identified as enhancing soybean tolerance to drought stress [3]. Therefore, there is a need to consider environmental stressors in future microbiome studies to highlight the potential of microbiome engineering for sustainable agriculture. Future research should focus on characterizing the functional roles of key microbial taxa and understanding their interactions with soybean genotypes under different environmental conditions. This will facilitate the development of microbial inoculants or genotype-specific management strategies to improve soybean productivity and resilience.

Overall, our findings emphasize the critical role of rhizosphere microbial communities in influencing soybean nodulation efficiency and plant performance. By utilizing the potential of the latest technological advancements, such as single-cell multiome and network biology, it can become relatively accessible to understand intricate plant-microbe interactions [57]. This information can aid in using beneficial microbial taxa, such as those enriched in high-nodulation genotypes, to offer potential solutions for enhancing sustainable agricultural practices.

## 4. Materials and Methods

### 4.1. Plant Materials and Growth Conditions

Seeds from four soybean genotypes (PI 458505, PI 603490, PI 605839 A, and PI 548400) obtained from the USDA Germplasm Resources Information Network (GRIN) were grown at the Rodney Foil Plant Science Research Center (33°28′ N, 88°47′ W), Mississippi State University, Rodney Foil Plant Science Research Center of Mississippi State University, Mississippi State, MI, USA. Three seeds were sown in each pot (85 cm height, 10 cm width) containing the sieved farm soil. A total of 20 plants (5 per genotype) were maintained under control conditions, and all the pots were fertilized with a controlled-release Osmocote (1.5 g, 14-14-14 N-P-K, Hummert International). Plant establishment and data collection were performed using the established protocols [58,59]. In brief, for plant physiological data, stomatal conductance was measured 14 days after stress imposition using an LI-600 between 10:00 a.m. and 12:00 p.m. on a sunny day on the third fully expanded trifoliate leaf. The leaf area was measured using an LI-3100C leaf area meter (LI-COR Biosciences, Lincoln, NE, USA). The root system was separated from the shoot, washed, and nodules traits were recorded. Shoot and root samples were dried at 75 °C for 72 h to determine biomass.

### 4.2. Rhizosphere Sample Collection

The same four genotypes were grown in custom-built root beds to determine genotype-driven rhizosphere microbial abundance with those contrasting nodulation genotypes. The rhizosphere soil samples from all four soybean genotypes were collected from the field-grown plants at the R1 stage. These samples were carefully placed into large Ziploc bags and transported to the laboratory. The samples were stored at 4 °C in a cold room to preserve microbial integrity until further processing. For DNA extraction, soil adhering closely to the plant roots was separated using sterile gloves and spatulas to minimize contamination and transferred into 50 mL falcon tubes. Each sample was labeled with the corresponding plant genotype for accurate identification.

### 4.3. DNA Isolation for 16S Amplicon Sequencing

A total of 0.20 g of soil per sample was used for DNA extraction using the DNeasy PowerSoil Pro Kit (Qiagen, Hilden, Germany) according to the manufacturer’s protocol. The extracted DNA was then quantified and assessed for quality using a NanoDrop 1000 Spectrophotometer (Thermo Scientific, Wilmington, DE, USA). Subsequent steps, including library preparation, quality control, and amplicon sequencing, were carried out at BGI Americas in Cambridge, MA, USA.

### 4.4. Taxonomic Abundance Analysis

The 16S amplicon sequencing data were analyzed using three biological replicates per sample. Demultiplexed sequences were processed through the Qiime2 pipeline [60], employing the DADA2 algorithm for quality filtering and denoising. Amplicon sequence variants (ASVs) generated were taxonomically classified by aligning them with the Silva 138 reference database at 99% similarity [61]. ASVs identified as “chloroplast”, “Archaea”, or “mitochondria” were excluded from further evaluation.

### 4.5. Microbial Diversity Analysis

Microbial diversity analyses were conducted using the “vegan (version V.2.6.6.1)” package [62] in R Statistical Software (v4.4.0; R Core Team, Vienna, Austria, 2024). Alpha diversity metrics, including Shannon, Chao1, and ACE indices, were compared using the Wilcoxon Rank Sum Test. Beta diversity was assessed with Bray–Curtis distance matrices, and principal coordinate analysis (PCoA) was utilized to visualize variations in bacterial community structure. Differences in bacterial community makeup among groups were evaluated using PERMANOVA (Adonis) with 999 permutations, considering a significance threshold of *p* < 0.05.

### 4.6. Statistical Data Analysis

Statistical analyses of the plant physiological data were performed using GraphPad Prism Statistical Analysis and Graphing Software (Version 10.2.0) (335). To evaluate differences in physiological responses, including stomatal conductance, number of nodules, nodule fresh weight, leaf area, shoot biomass, and root biomass among the four soybean genotypes (PI 458505, PI 603490, PI 605839 A, and PI 548400), the means and *p*-values were calculated using both a one-sample *t*-test and the Wilcoxon test. Data are presented as the mean of five replicates ± standard error (SE). Statistical significance was indicated by asterisks on the vertical bars with the following thresholds: * *p* < 0.05, ** *p* < 0.005, *** *p* < 0.0005, and **** *p* < 0.00005, respectively, while non-significant differences were indicated by ‘ns’. Additionally, statistical analyses of microbiome data were performed using R version 4.2.1. Alpha diversity indices, including ACE, Chao1, Observed OTUs, and Shannon diversity, were calculated using the ‘vegan’ package. Differences in alpha diversity between high- and low-nodulation groups were assessed using the non-parametric Kruskal–Wallis Rank Sum Test. Beta diversity was analyzed using principal coordinate analysis (PCoA) based on Bray–Curtis dissimilarity, and differences in community composition were tested using PERMANOVA with 999 permutations. Venn diagrams were generated using the ‘VennDiagram (Mioeco version 1.7.1)’ package to visualize shared and unique taxa between groups. For all statistical tests, a significance threshold of *p* < 0.05 was applied.

## 5. Conclusions

Our findings demonstrate that soybean genotypes significantly influence the diversity and composition of rhizosphere microbial communities and are correlated with nodulation capacity. *Acidobacteria* were found to be more abundant in genotypes with high nodulation, whereas Firmicutes and *Planctomycetota* were preferred in genotypes with low nodulation. *Proteobacteria* were consistently dominant across all genotypes. Different microbial communities associated with nodulation performance were identified by alpha and beta diversity studies, indicating genotype-specific recruitment of rhizosphere microbiota. These results highlight the potential for genotype-driven approaches to harness beneficial microbial communities for improving soybean nodulation and productivity. However, further research is needed to elucidate the functional roles of key microbial taxa and their interactions with soybean genotypes under varying environmental conditions. Advanced tools such as single-cell multiomics and network analysis could provide deeper insights into the mechanisms underlying plant-microbe interactions. To improve soybean resilience and sustainable agricultural outcomes, this knowledge might direct the development of genotype-specific management techniques or customized microbial inoculants.

## Figures and Tables

**Table 1:** Group classification and rhizosphere soil sample IDs for corresponding soybean genotypes.

Group Name	Soil Sample ID	Soybean Genotype ID	Amount of Soil for DNA Isolation
**G1 (High nodulation group)**	SY105	PI 458505	200 mg
	SY107	PI 603490	200 mg
**G2 (Low nodulation group)**	SY112	PI 605839 A	200 mg
	SY209	PI 548400	200 mg

## Data Availability

The original contributions presented in the study are included in the article. Further inquiries can be directed to the corresponding author.
